# Supplementary material for: Anti-Pathogenic Properties of the Combination of a T3SS Inhibitory Halogenated Pyrrolidone with C-30 Furanone
Source: Molecules. 2021 Dec 16;26(24):7635. doi: 10.3390/molecules26247635 (PMC8707098; doi:10.3390/molecules26247635)
Supplement: Supplementary file 1 [file molecules-26-07635-s001.zip › molecules-1486642-supplementary.pdf]

Supplementary Materials

Anti-pathogenic properties of the combination of a T3SS inhibitory halogenated pyrrolidone with C-30 furanone

Nelly Araceli Aburto-Rodríguez, Naybi Muñoz-Cázares, Víctor Alberto Castro-Torres, Bertha González-Pedrajo, Miguel Díaz-Guerrero, Rodolfo García-Contreras, Héctor Quezada, Israel Castillo-Juárez and Mariano Martínez-Vázquez.

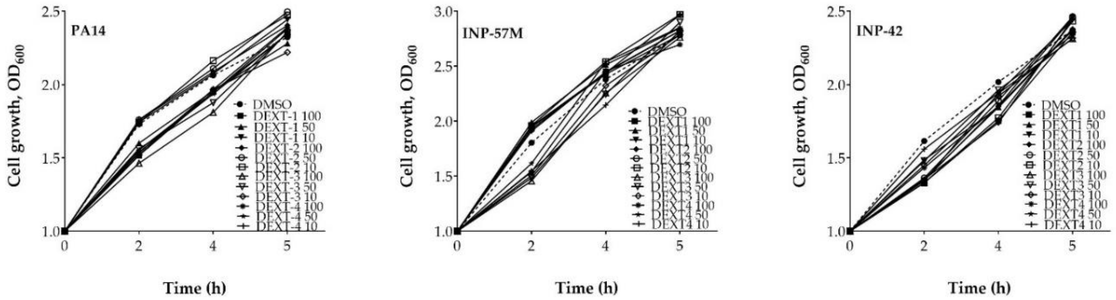

Figure S1. Effect of the pyrrolidones DEXT 1-4 on the growth of *P. aeruginosa* strains. PA14 wild type and the clinical isolates INP-57M and INP-42.

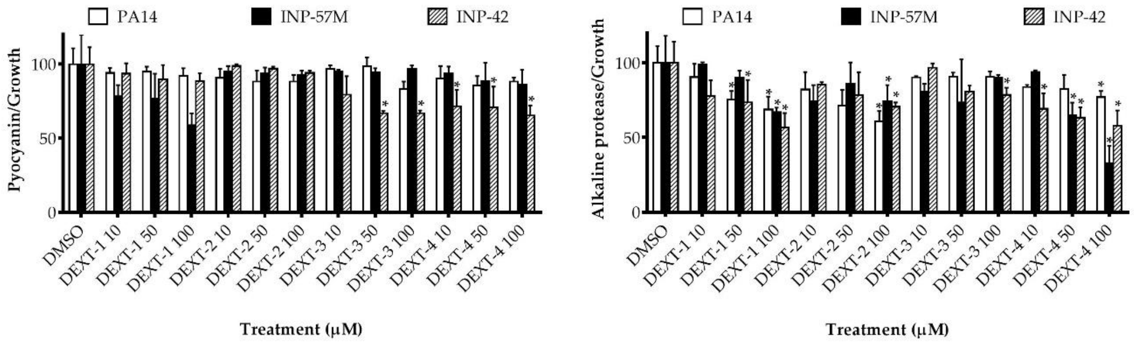

Figure S2. Effect of pyrrolidones DEXT 1-4 on pyocyanin production and alkaline protease activity. PA14 wild type and the clinical isolates INP-57M and INP-42. Values are presented as means ± SD. The Student's t-test was used to calculate the differences between two mean values ($p < 0.05$).

Spectroscopic data for DEXT 1-4 pyrrolidone

The assignments of the NMR data of all the synthesized compounds are shown in the results and discussion section of the article.

Ethyl 4-hydroxy-5-oxo-1,2-diphenyl-2,5-dihydro-1*H*-pyrrole-3-carboxylate (DEXT-1).

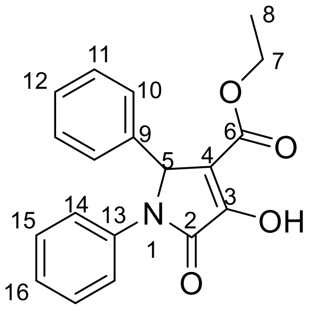

M.p. 152-153 °C

MS, *m/z* (DART +): 324.12214

Anal. Calcd for $^{12}C_{19}{}^{1}H_{18}{}^{14}N_{1}{}^{16}O_{4}$

NMR ^{1}H (300 MHz, $CDCl_3$) δ 7.51-7.43 (m, 2H), 7.30-7.18 (m, 7H), 7.12-7-18 (m, 1H), 5.73(s, 1H), 4.18 (q, *J*=7.1Hz, 2H), 1.17 (t, *J*=7.1 Hz, 3H)

NMR ^{13}C (75 MHz, $CDCl_3$) δ 165.21, 163.01, 156.58, 136.39, 135.21, 129.05, 128.68, 128.61, 127.64, 125.93, 122.39, 113.28, 61.69, 61.34, 14.02

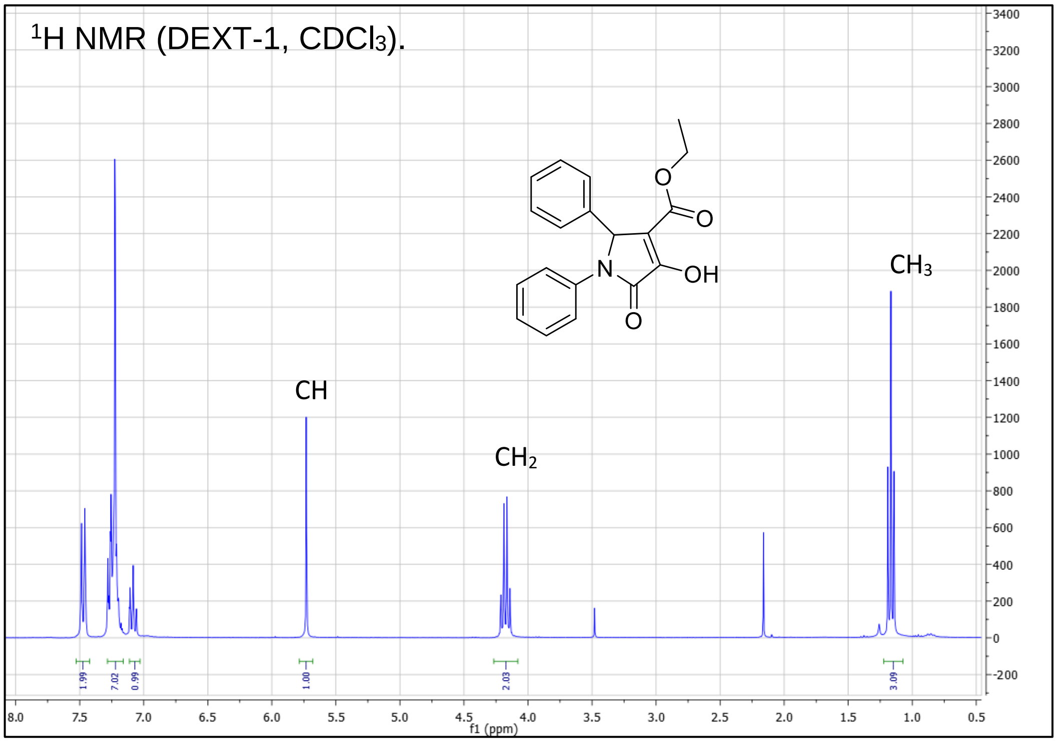

1H NMR (DEXT-1, CDCl3).
CH
CH2
CH3
O
OH
N
1.99
7.02
0.99
1.00
2.03
3.09
f1 (ppm)

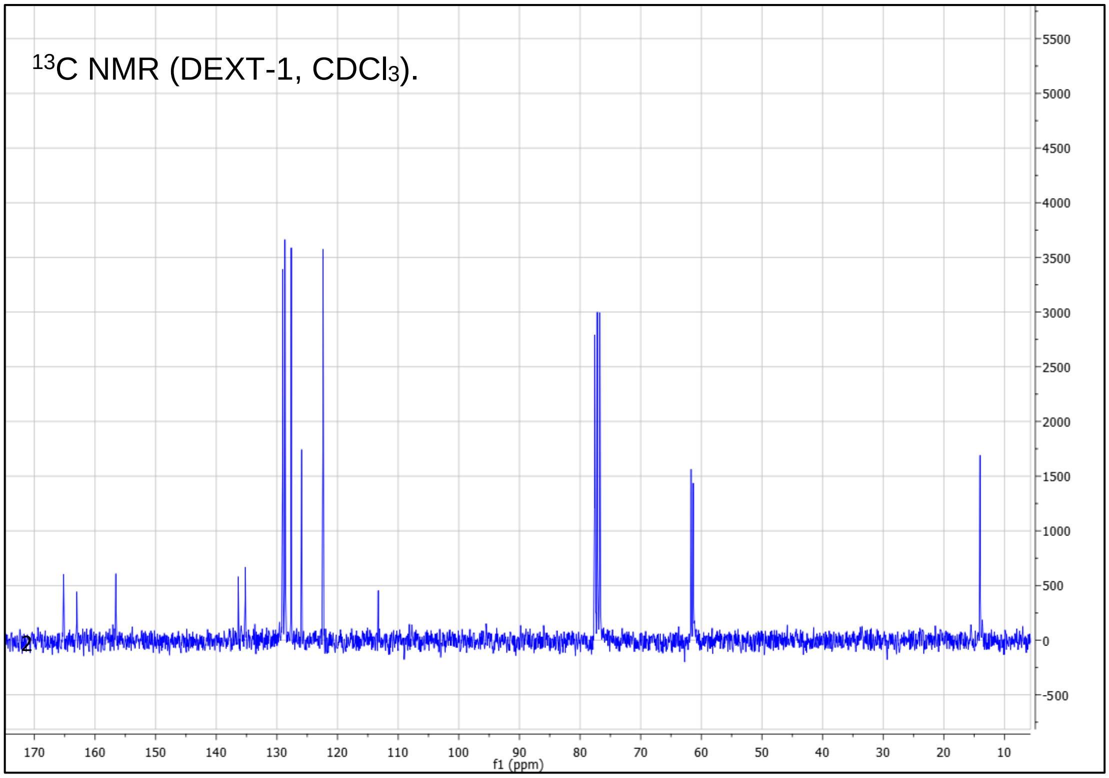

13C NMR (DEXT-1, CDCl3).
f1 (ppm)

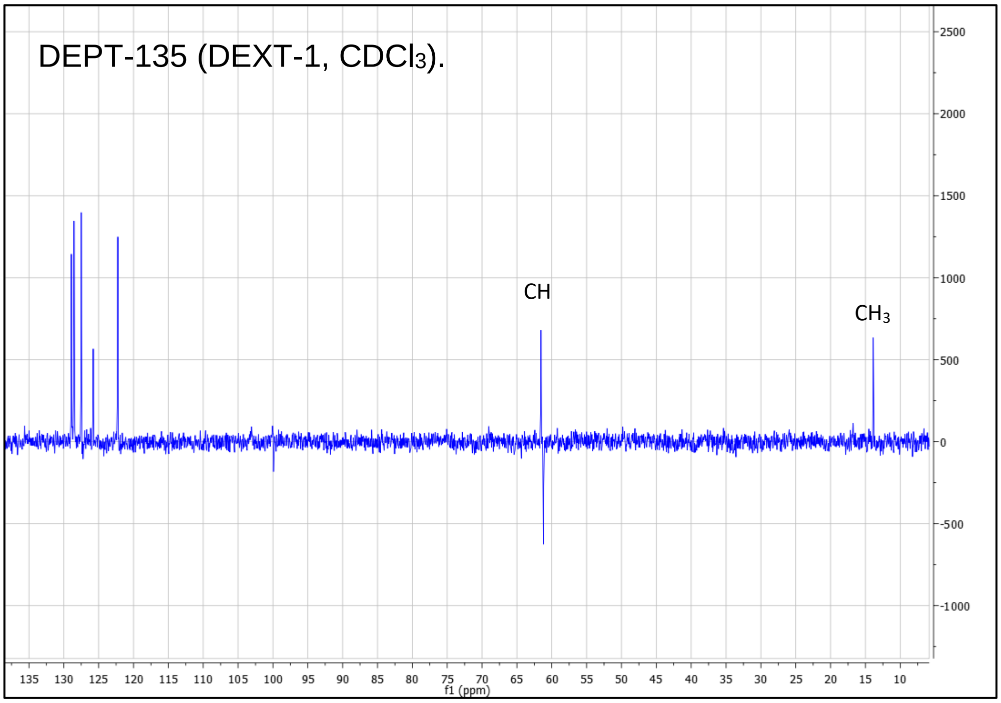
DEPT-135 (DEXT-1, CDCl3).
CH
CH3
f1 (ppm)

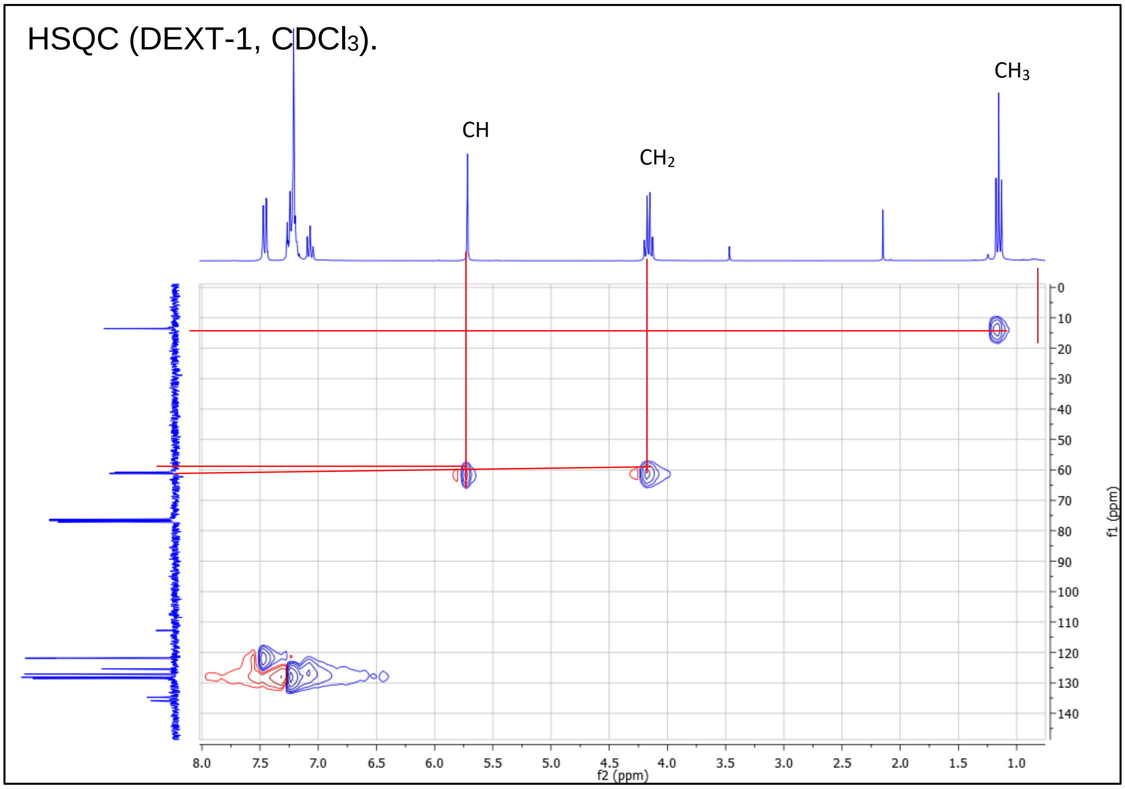
HSQC (DEXT-1, CDCl3).
CH
CH2
CH3
f2 (ppm)
f1 (ppm)

Infrared spectrum (DEXT-1)

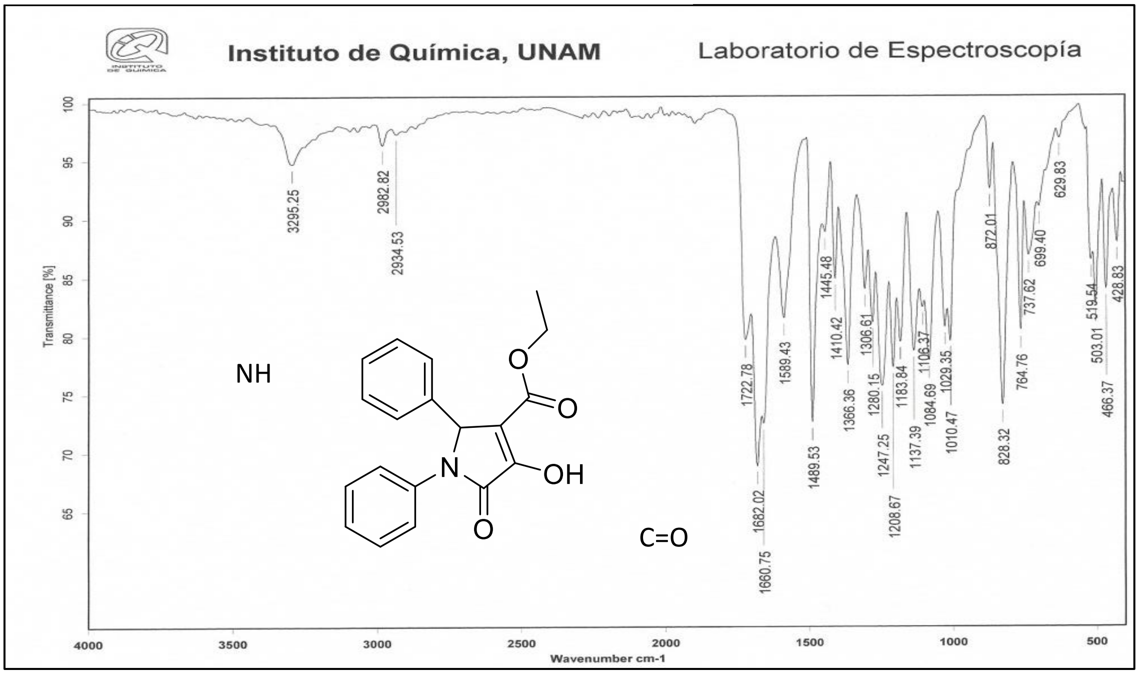

Mass spectrum (DART+) of DEXT-1

Data:1414 DX1
Sample Name:Dr Martinez Mariano 2016
Description:
Ionization Mode:ESI+
History:Determine m/z[Peak Detect[Centroid,30,Area];Correct Base[5.0%]];Correct Base[5.0%];Average(MS[1] 1..1)

Acquired:4/21/2016 4:04:19 PM
Operator:AccuTOF
Mass Calibration data:Cal_Peg_600
Created:4/21/2016 4:51:59 PM
Created by:AccuTOF

Charge number:1
Tolerance:3.00(mmu)
Unsaturation Number:0.0 .. 30.0 (Fraction:Both)
Element:^{12}C:0 .. 50, ^{1}H:0 .. 100, ^{14}N:0 .. 3, ^{16}O:0 .. 5

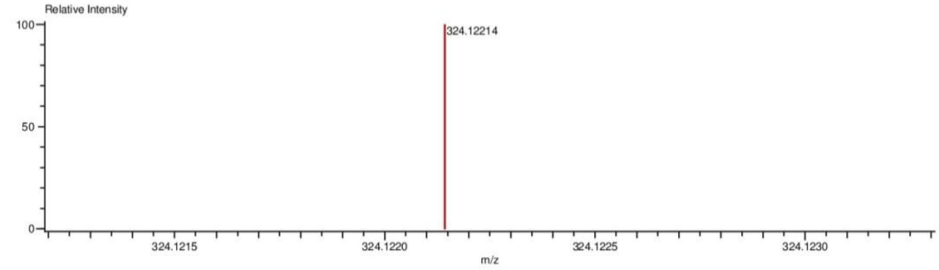

Mass	Intensity	Calc. Mass	Mass Difference (mmu)	Mass Difference (ppm)	Possible Formula	Unsaturation Number
324.12214	23630.00	324.12358	-1.44	-4.44	$^{12}C_{19}{}^{1}H_{18}{}^{14}N_{1}{}^{16}O_{4}$	11.5

Ethyl2-(4-chlorophenyl)-4-hydroxy-5-oxo-1-phenyl-2,5-dihydro-1*H*-pyrrole-3-carboxylate (DEXT-2).

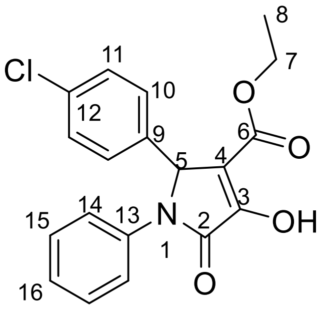

M.p. 165-167 ^{o}C

MS, *m/z* (DART +): 358.08585

Anal. Calcd for $^{12}C_{19}{}^{1}H_{17}{}^{35}Cl_{1}{}^{14}N_{1}{}^{16}O_{4}$

NMR ^{1}H (300 MHz, $CDCl_3$) δ 7.49-7.39 (m, 2H), 7.34-7.06 (m, 7H), 5.72 (s, 1H), 4.20(c, *J*=7.1Hz, 2H), 1.20 (t, *J*=7.1 Hz, 3H)

NMR ^{13}C (75 MHz, $CDCl_3$) δ 164.98, 162.87, 156.66, 136.12, 134.44, 133.92, 129.20, 129.00, 126.16, 122.41, 112.87, 61.50, 60.99, 14. 11

^1H NMR (DEXT-2, $CDCl_3$).

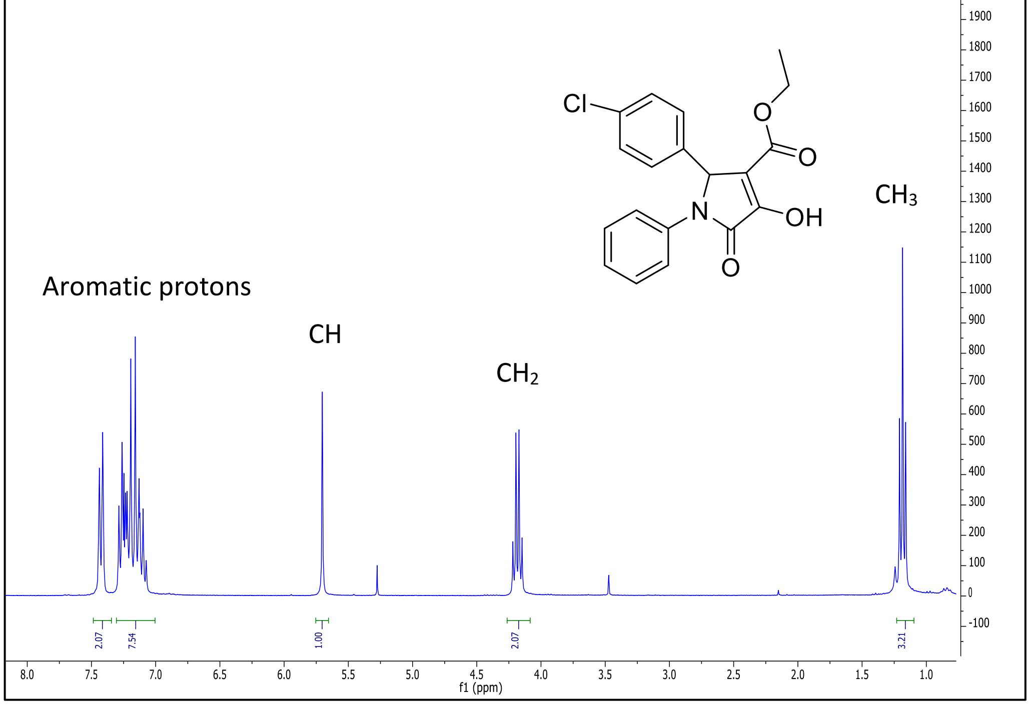

^{13}C NMR (DEXT-2, $CDCl_3$).

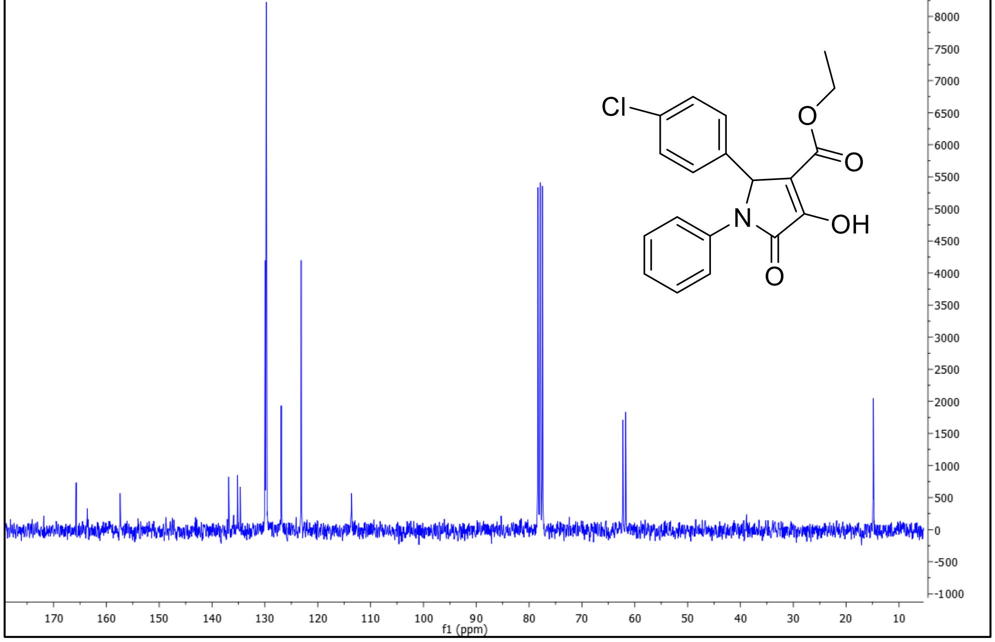

DEPT-135 (DEXT-2, CDCl3).

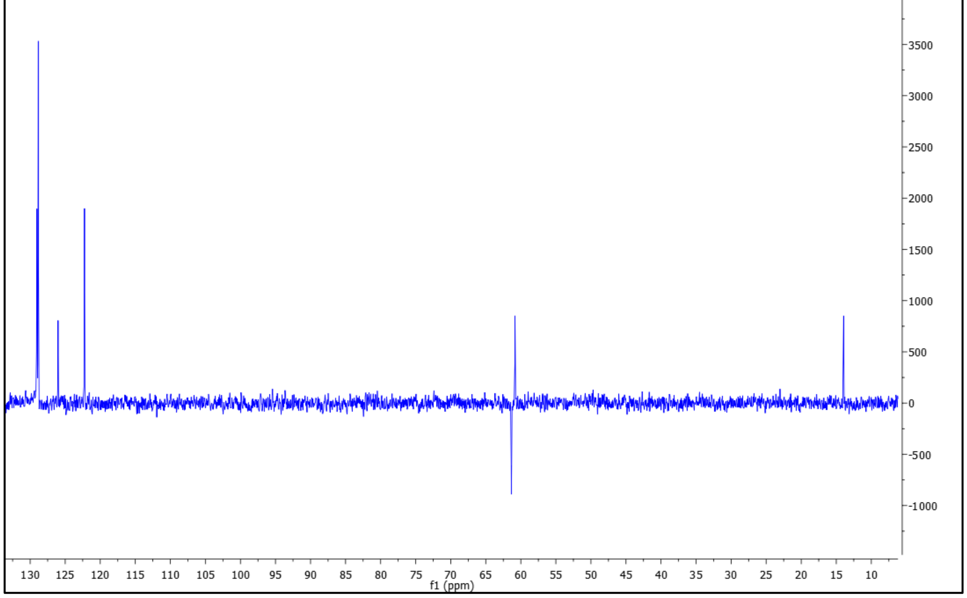

HSQC spectrum of DEXT-2 in $CDCl_3$.

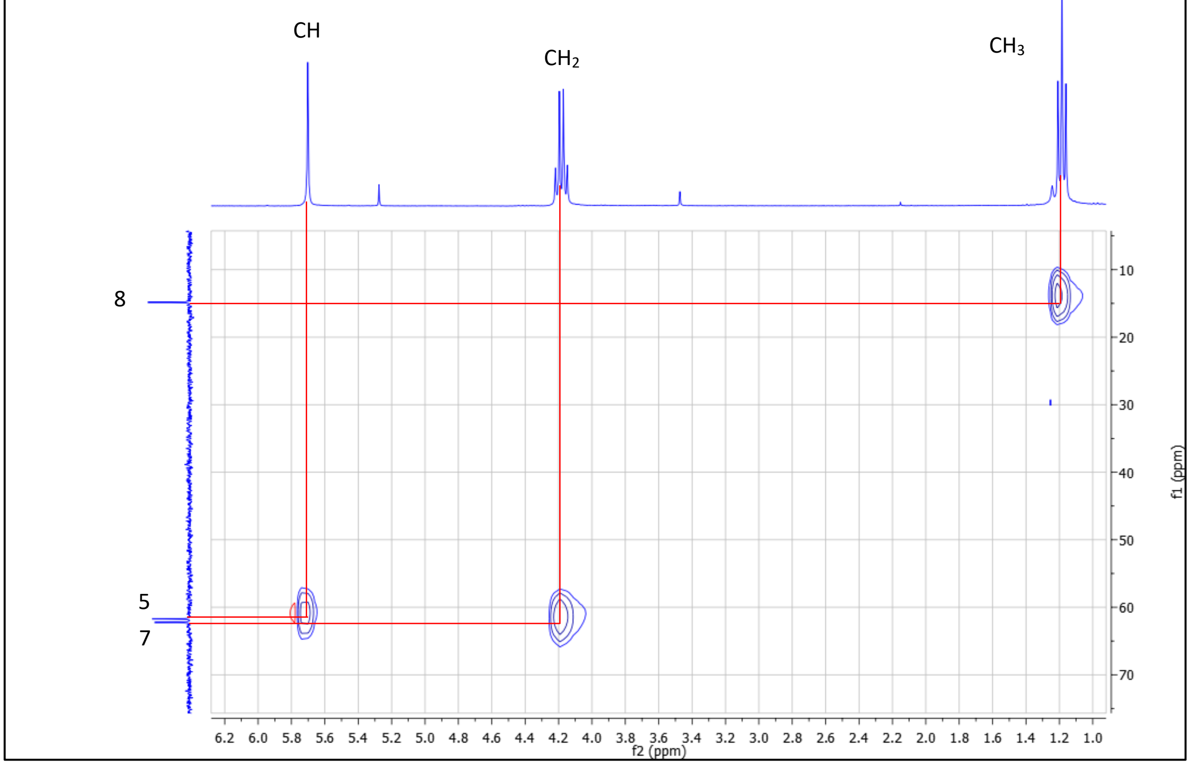

Infrared spectrum (DEXT-2).

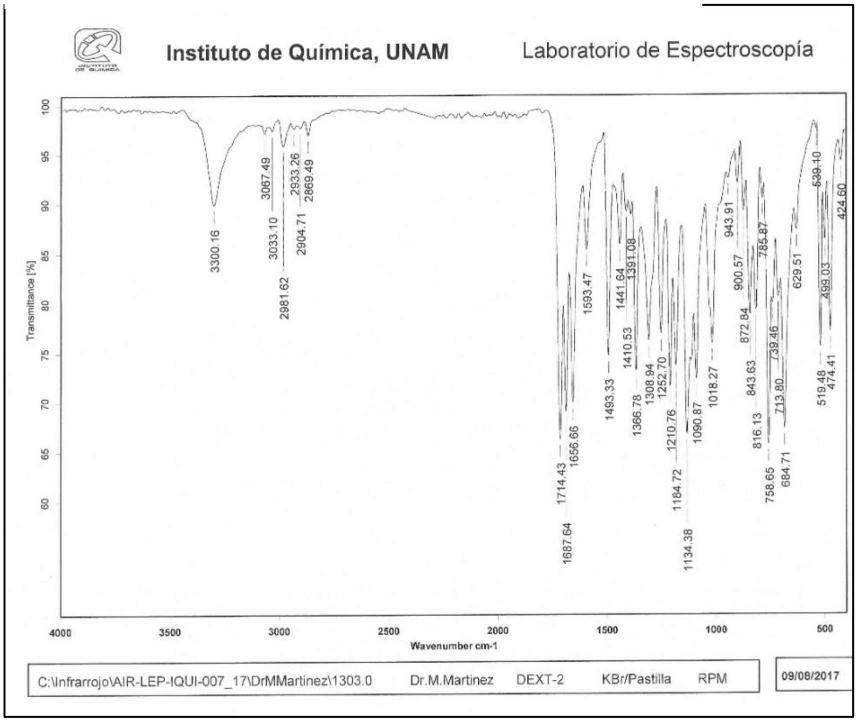

Mass spectrum (DART+) of DEXT-2.

Data:1482 DX2
Sample Name:Dr Martinez Mariano 2016
Description:
Ionization Mode:ESI+
History:Determine m/z[Peak Detect[Centroid,30,Area];Correct Base[5.0%]];Correct Base[5.0%];Average(MS[1] 1..1)

Acquired:4/26/2016 3:02:26 PM
Operator:AccuTOF
Mass Calibration data:Cal_Peg_600
Created:4/26/2016 3:46:02 PM
Created by:AccuTOF

Charge number:1
Tolerance:3.00(mmu)
Unsaturation Number:0.0 .. 30.0 (Fraction:Both)
Element:^{12}C:0 .. 20, ^{1}H:0 .. 20, ^{35}Cl:0 .. 1, ^{14}N:0 .. 2, ^{16}O:0 .. 10

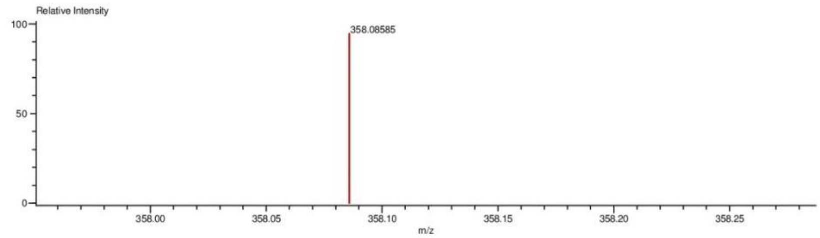

Mass	Intensity	Calc. Mass	Mass Difference (mmu)	Mass Difference (ppm)	Possible Formula	Unsaturation Number
358.08585	2999.23	358.08461	1.24	3.46	$^{12}C_{19}{}^{1}H_{17}{}^{35}Cl_{1}{}^{14}N_{1}{}^{16}O_{4}$	11.5

Ethyl 1-(4-bromophenyl)-4-hydroxy-5-oxo-2-phenyl-2,5-dihydro-1*H*-pyrrole-3-carboxylate (DEXT-3).

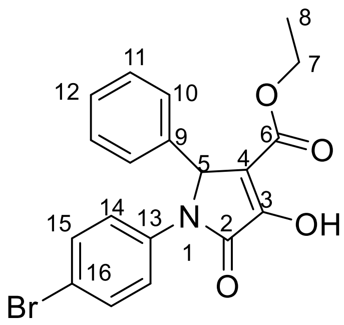

M.p. 144-147 °C

MS, *m/z* (DART +): 404.03405

Anal. Calcd for $^{12}C_{19}{}^{1}H_{17}{}^{81}Br_{1}{}^{14}N_{1}{}^{16}O_{4}$

NMR ^{1}H (300 MHz, $CDCl_3$) δ 7.51-7.53 (m, 2H), 7.3-7.18 (m, 6H), 7.13-7.03 (m, 1H), 5.73 (s, 1H), 4.18 (c, *J*= 7.1 Hz, 2H), 1.17 (t, *J*= 7.1 Hz, 3H)

NMR ^{13}C (75 MHz, $CDCl_3$) δ 165.21, 163.01, 156.57, 136.39, 135.21, 129.05, 128.68, 128.61, 127.64, 125.93, 122.39, 122.39, 61.69, 61.34, 14.03

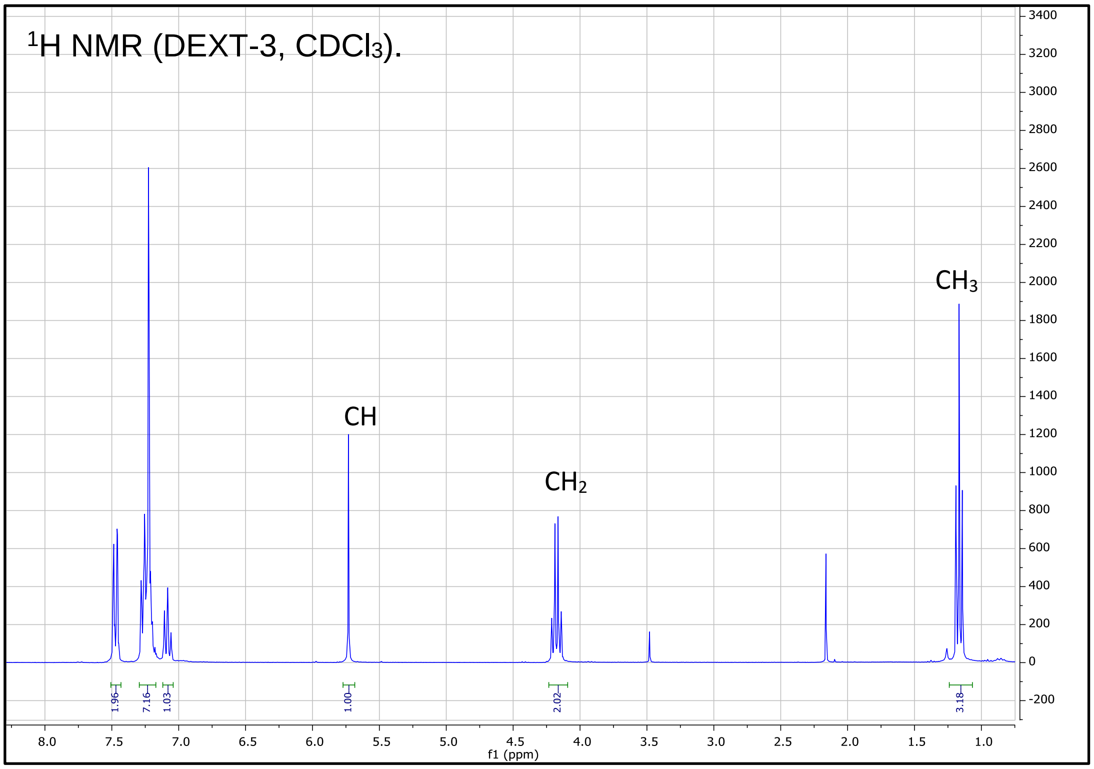

^{1}H NMR (DEXT-3, CDCl3).
CH
CH2
CH3
1.96
7.16
1.03
1.00
2.02
3.18
f1 (ppm)

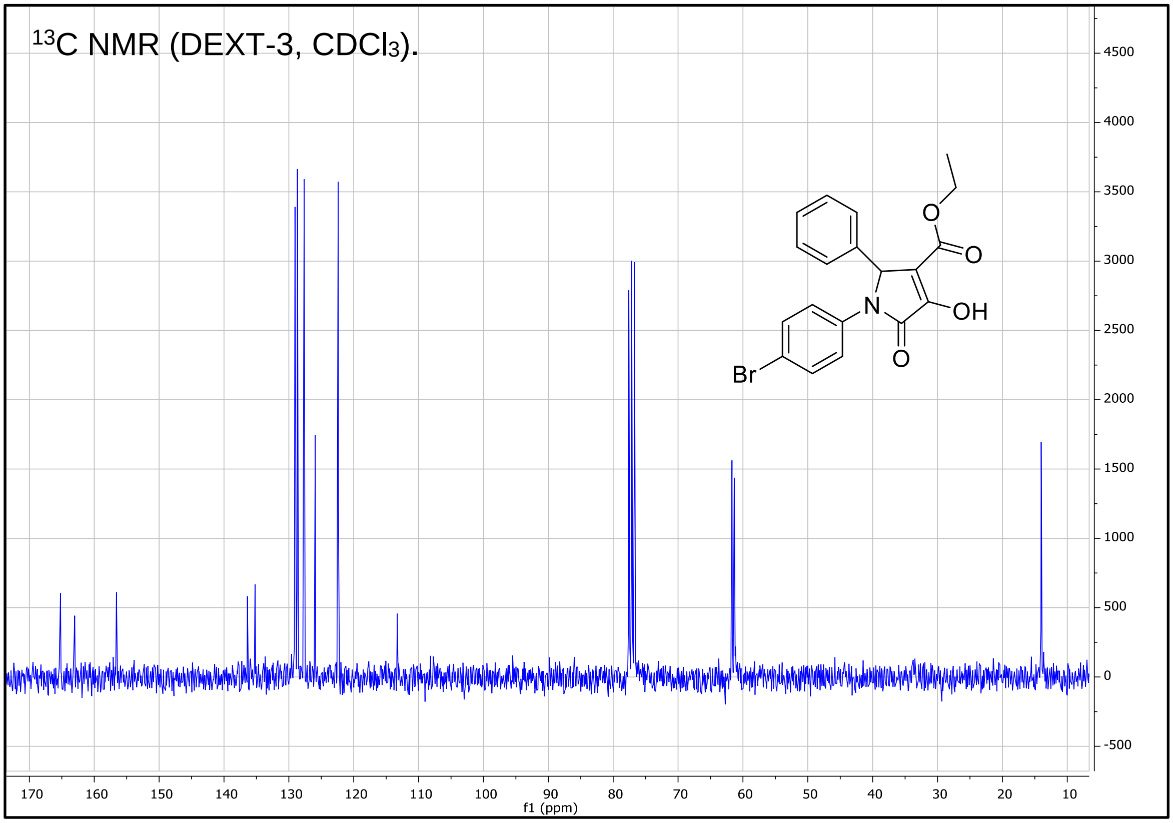

^{13}C NMR (DEXT-3, CDCl3).
Br
N
O
OH
f1 (ppm)

DEPT-135 spectrum of DEXT-3 in $CDCl_3$.

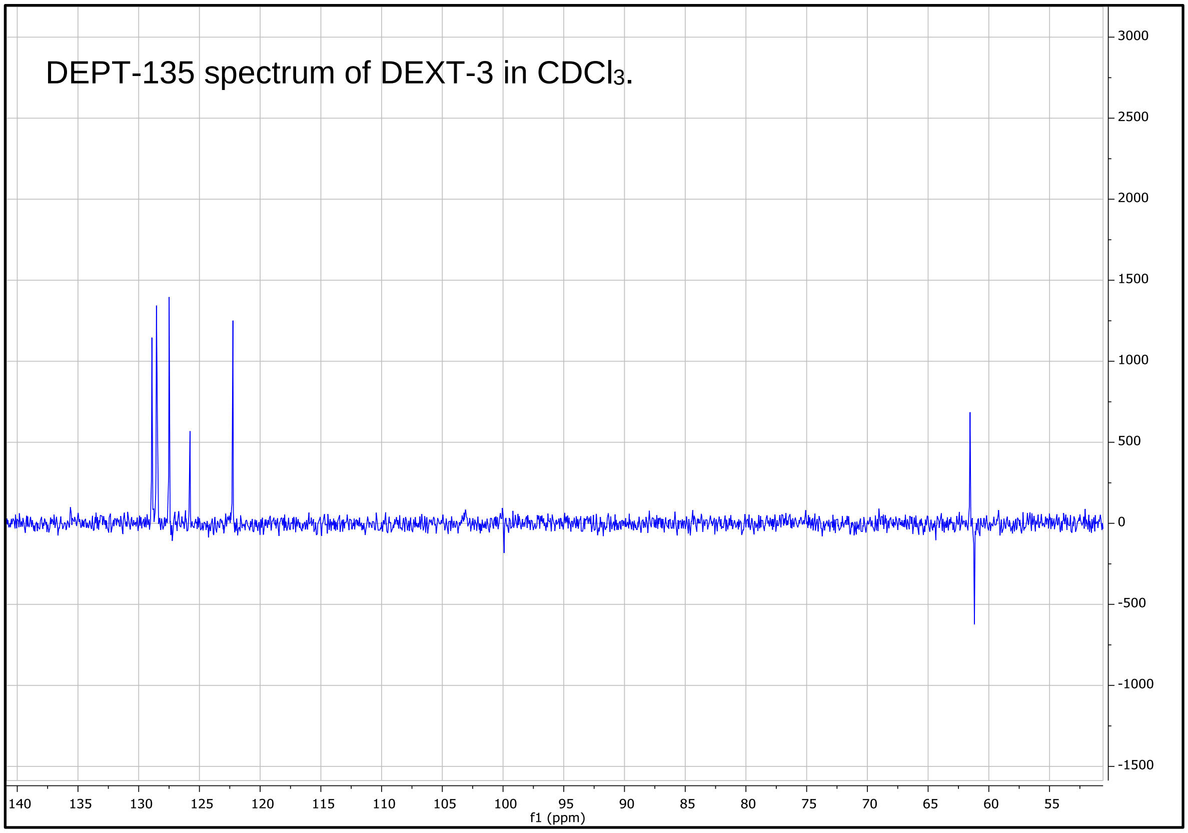

HSQC spectrum of DEXT-3 in $CDCl_3$.

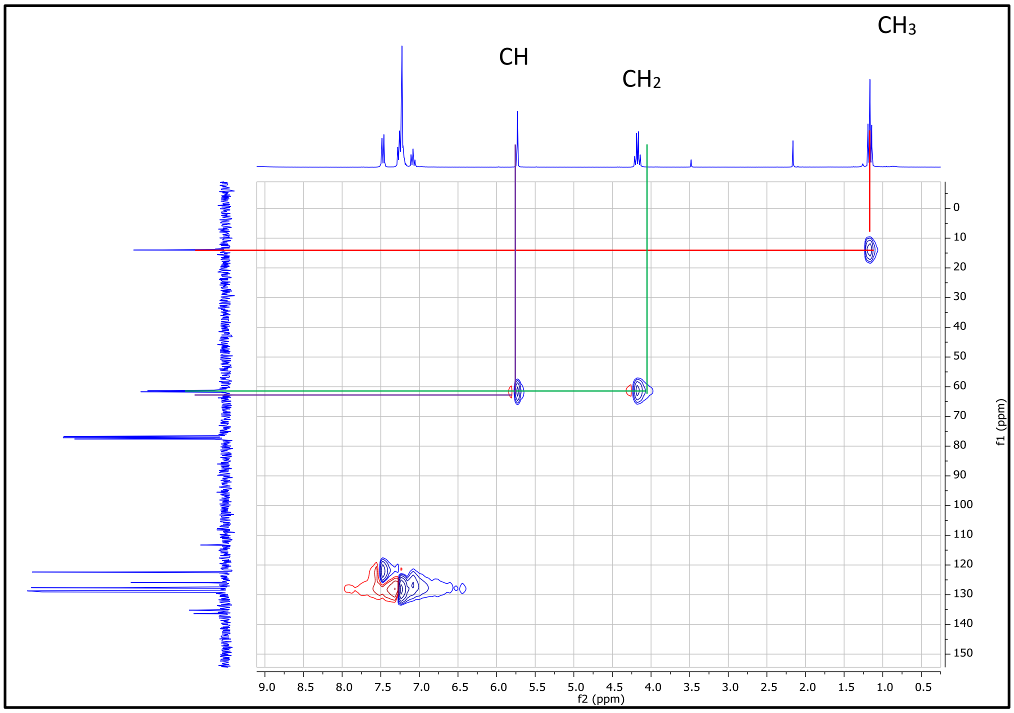

Infrared spectrum (DEXT-3).

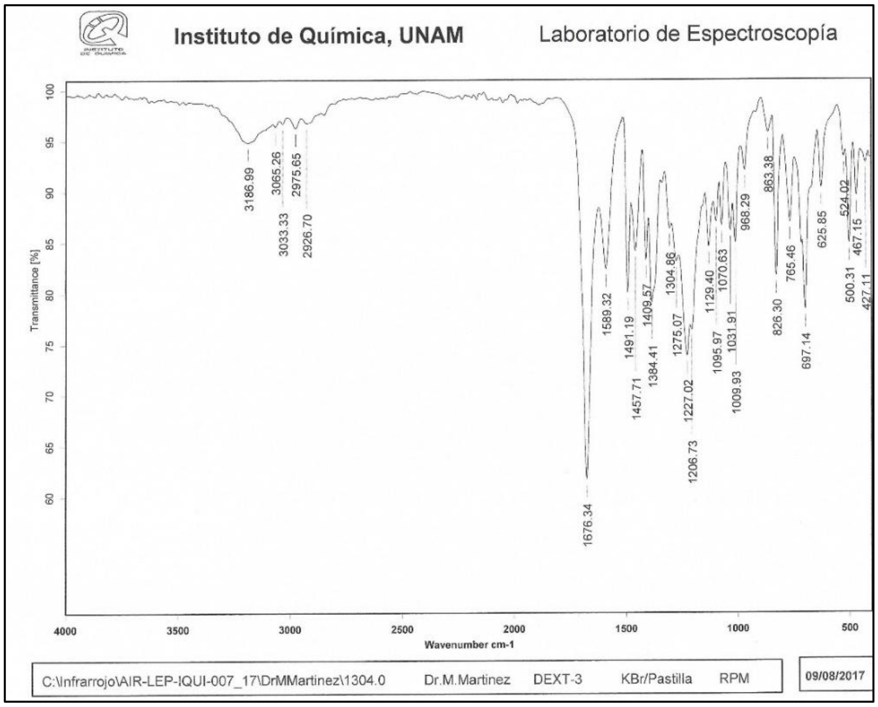

Mass spectrum (DART+) of DEXT-3

Data:1823 L2A 17-21
Sample Name:Dr Martinez Mariano 2016
Description:
Ionization Mode:ESI+
History:Determine m/z[Peak Detect[Centroid,30,Area];Correct Base[10.0%]];Correct Base[5.0%];Average(MS[1] 2)

Acquired:5/16/2016 10:05:57 AM
Operator:AccuTOF
Mass Calibration data:Cal PEG 600
Created:5/16/2016 10:33:23 AM
Created by:AccuTOF

Charge number:1
Tolerance:3.00(mmu)
Unsaturation Number:0.0 .. 50.0 (Fraction:Both)
Element:^{12}C:0 .. 20, ^{1}H:0 .. 20, ^{79}Br:0 .. 1, ^{81}Br:0 .. 1, ^{14}N:0 .. 2, ^{16}O:0 .. 5

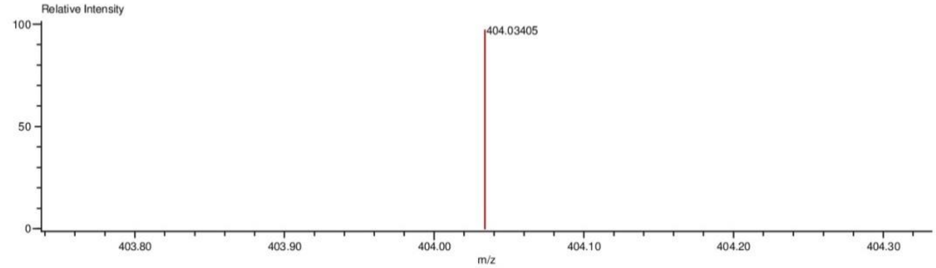

Mass	Intensity	Calc. Mass	Mass Difference (mmu)	Mass Difference (ppm)	Possible Formula	Unsaturation Number
404.03405	13015.50	404.03205	2.00	4.95	$^{12}C_{19}{}^{1}H_{17}{}^{81}Br_{1}{}^{14}N_{1}{}^{16}O_{4}$	11.5

Ethyl 1-(4-fluorophenyl)-4-hydroxy-5-oxo-2-phenyl-2,5-dihydro-1*H*-pyrrole-3-carboxylate (DEXT-4).

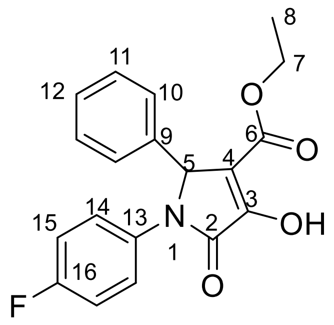

M.p. 157-160 ^{o}C

MS, *m/z* (DART +): 376.07514

Anal. Calcd for $^{12}C_{19}{}^{1}H_{16}{}^{35}Cl_{1}{}^{19}F_{1}{}^{14}N_{1}{}^{16}O_{4}$

NMR ^{1}H (300 MHz, $CDCl_3$) δ 7.46-7.31 (m, 2H), 7.29-7.19 (m, 2H), 7.14 (d, *J*= 8.4 Hz, 2H), 6.97 (t, *J*= 8.6 Hz, 2H), 5.65 (s, 1H), 4.20 (c, *J*= 7.1 Hz, 2H), 1.19 (t, *J*= 7.1 Hz, 3H)

NMR ^{13}C (75 MHz, $CDCl_3$) δ 165.02, 162.17, 158.90, 156.84, 134.65, 133.69, 132.13, 129.11, 128.98, 124.50, 124.40, 116.30, 116.0, 61.56, 61.36, 14.12

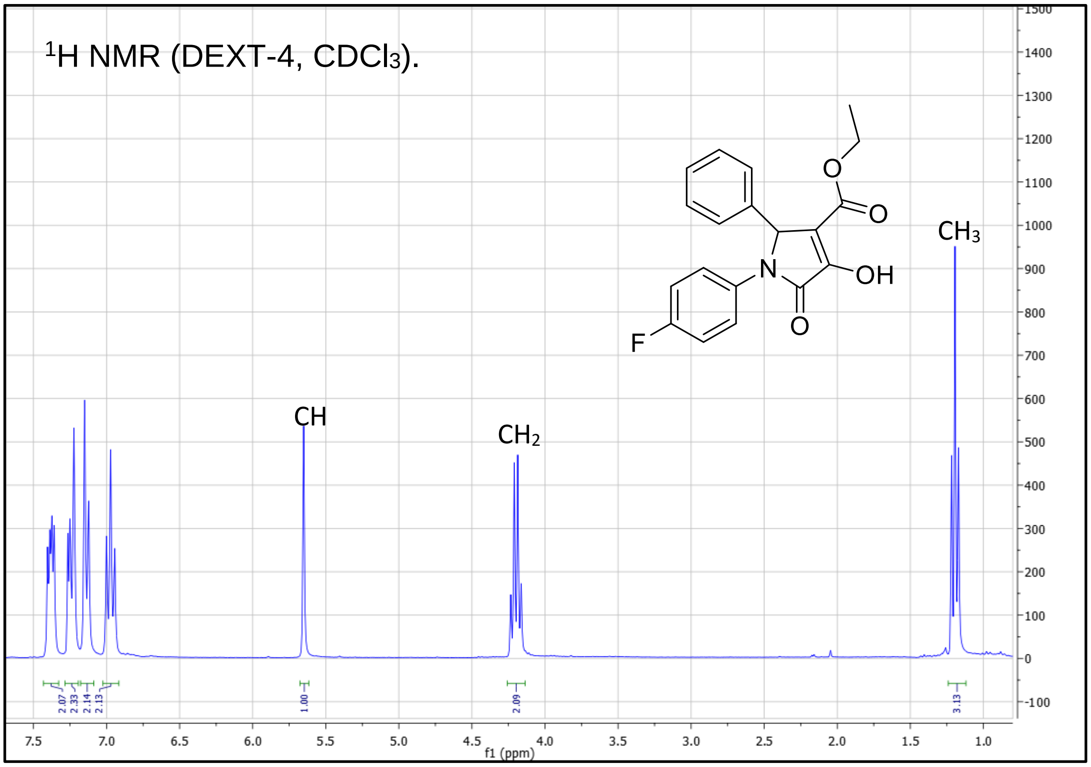

1H NMR (DEXT-4, CDCl3).
CH3
CH
CH2
OH
O
O
O
N
F
f1 (ppm)

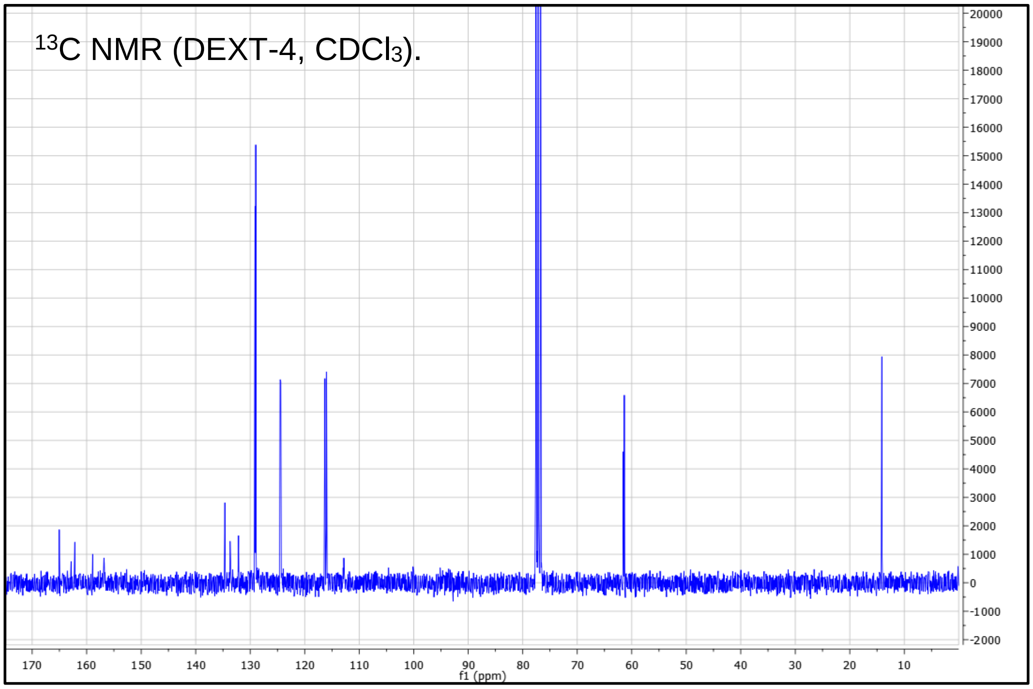

13C NMR (DEXT-4, CDCl3).
f1 (ppm)

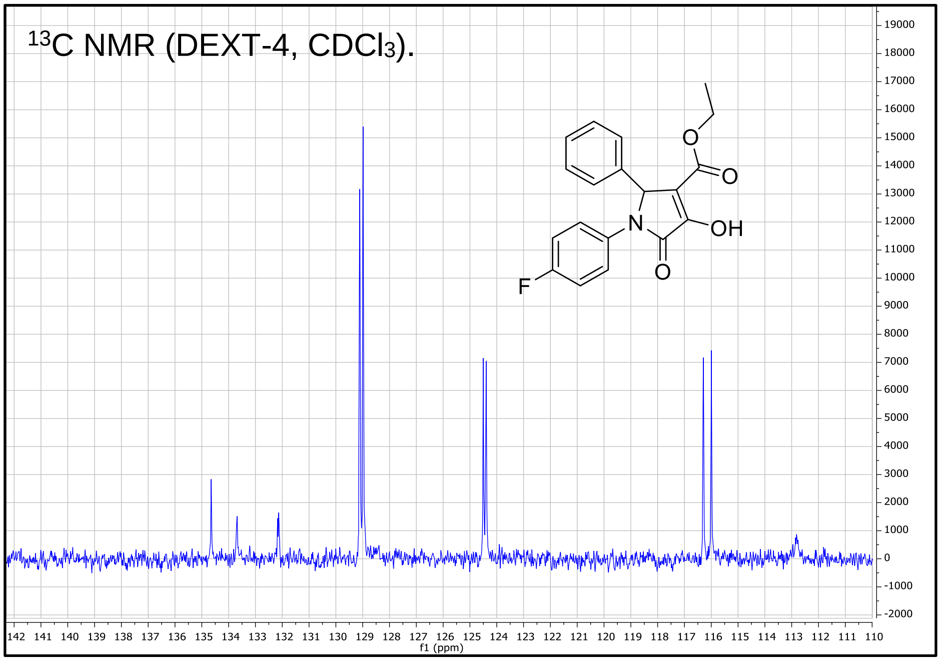

^{13}C NMR (DEXT-4, $CDCl_3$).

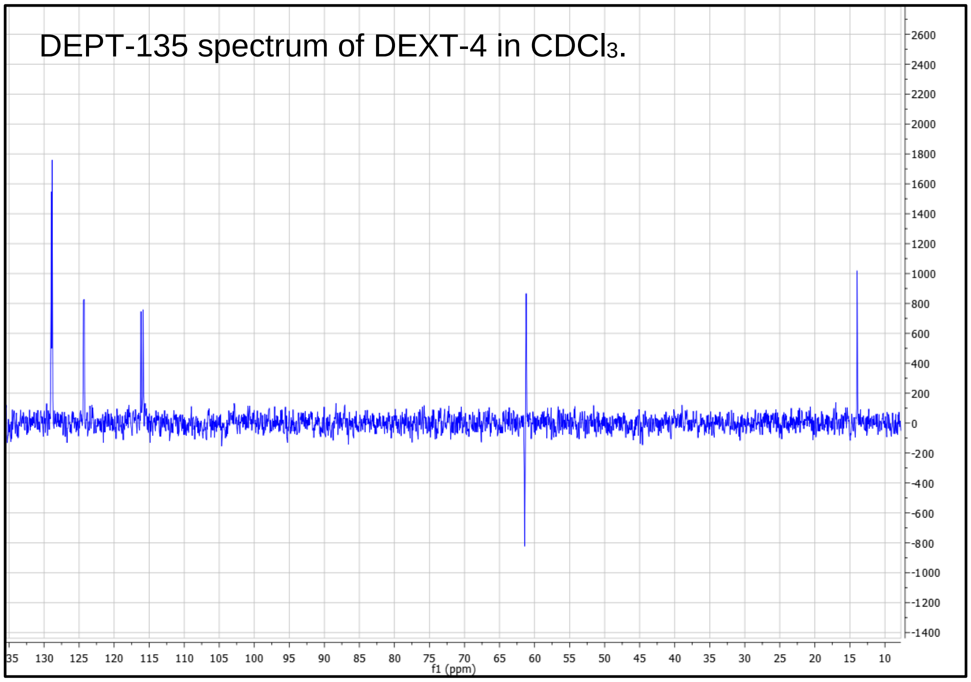

DEPT-135 spectrum of DEXT-4 in $CDCl_3$.

HSQC spectrum of DEXT-4 in $CDCl_3$.

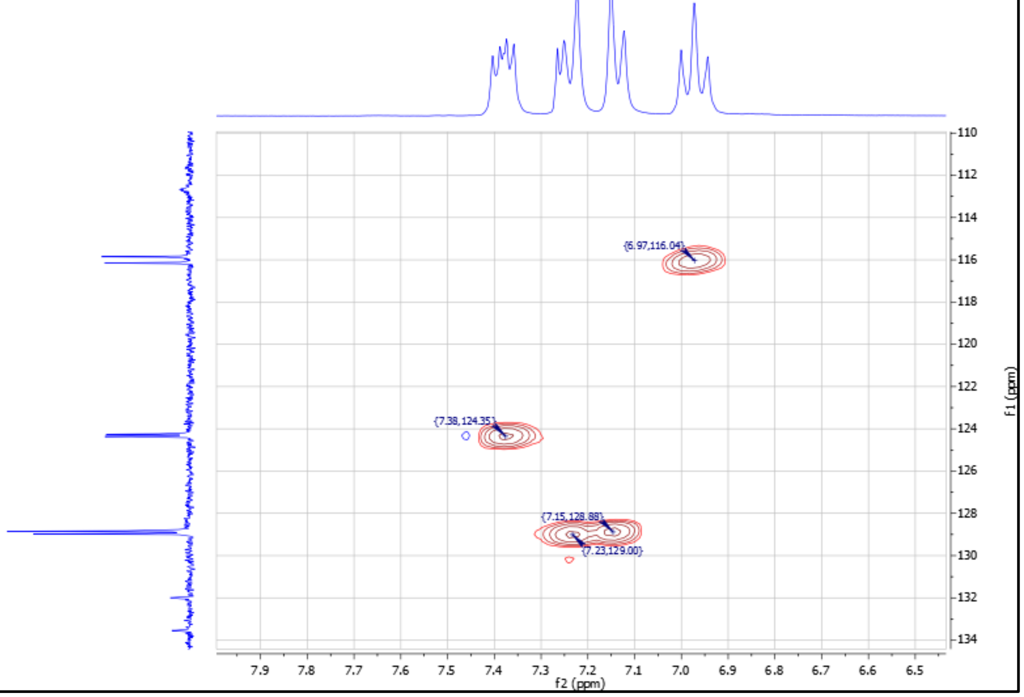

HMBC spectrum of DEXT-4 in $CDCl_3$.

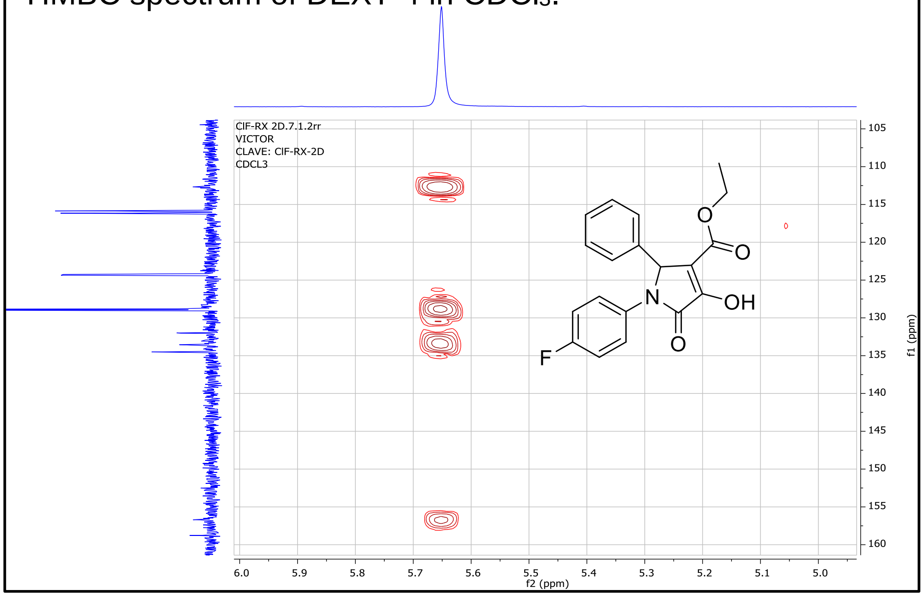

Infrared spectrum of DEXT-4

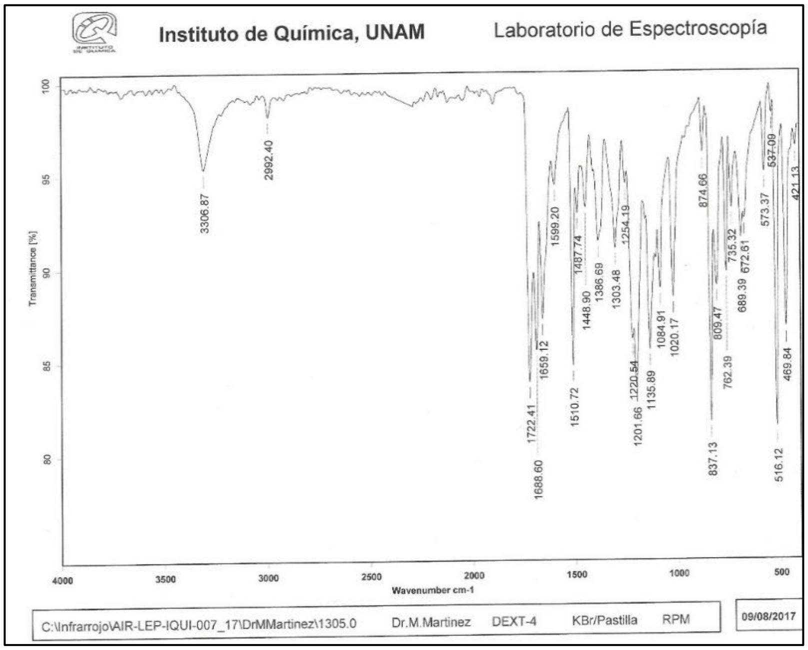

Mass spectrum (DART+) of DEXT-4.

Data:2336 IsQ RX POCl3
Sample Name:Dr Martinez Mariano 2016
Description:
Ionization Mode:ESI+
History:Determine m/z[Peak Detect[Centroid,30,Area];Correct Base[10.0%]];Correct Base[5.0%];Average(MS[1] 6..6)

Acquired:6/15/2016 12:36:15 PM
Operator:AccuTOF
Mass Calibration data:Cal_Peg_600
Created:6/15/2016 1:11:49 PM
Created by:AccuTOF

Charge number:1
Tolerance:3.00(mmu)
Unsaturation Number:0.0 .. 20.0 (Fraction:.5)
Element:^{12}C:0 .. 20, ^{1}H:0 .. 20, ^{35}Cl:1 .. 1, ^{19}F:0 .. 1, ^{14}N:0 .. 2, ^{16}O:1 .. 5

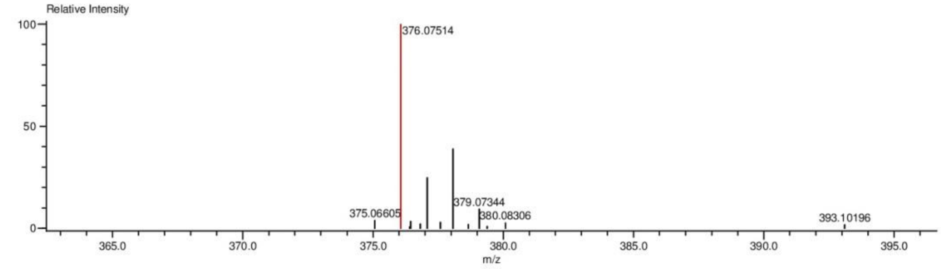

Mass	Intensity	Calc. Mass	Mass Difference (mmu)	Mass Difference (ppm)	Possible Formula	Unsaturation Number
376.07514	30014.25	376.07519	-0.05	-0.13	$^{12}C_{19}{}^{1}H_{16}{}^{35}Cl_{1}{}^{19}F_{1}{}^{14}N_{1}{}^{16}O_{4}$	11.5
